# Endoscopic grading of gastric intestinal metaplasia and microvascular pattern for assessing gastric cancer risk: a prospective study

**DOI:** 10.1080/07853890.2026.2668887

**Published:** 2026-05-10

**Authors:** Xueling Zhang, Jinxin Shi, Weijia Wang, Haotian Chen, Qian Li, Yixuan Wu, Yue Wu, Guangzhi Shi, Qinsheng Zhang, Wei Wen, Peilin Cui

**Affiliations:** ^a^ International Medical Services Department, Beijing Tiantan Hospital, Capital Medical University, Beijing, China; ^b^ Department of Intensive Care Unit, Beijing Tiantan Hospital, Capital Medical University, Beijing, China; ^c^ Department of Diagnosis and Treatment Center for Digestive Diseases of Henan Province Hospital of Traditional Chinese Medicine, Zhengzhou, Henan, China; ^d^ Department of Endoscopy Center, The Second Affiliated Hospital of Nanjing University of Chinese Medicine, Nanjing, Jiangsu, China

**Keywords:** Microvascular pattern, EGGIM, gastric intestinal metaplasia, OLGIM, gastric cancer

## Abstract

**Background and Aims:**

The endoscopic grading of gastric intestinal metaplasia (EGGIM) has not been comprehensively validated. Microvascular (MV) pattern abnormalities are critical for diagnosing gastric neoplastic transformation. The present study aims to validate the diagnostic accuracy of EGGIM for identifying high-risk GIM patients and to develop a modified EGGIM model incorporating microvascular pattern, evaluating its diagnostic performance for risk stratification of GC development in GIM patients.

**Methods:**

All patients underwent EGGIM scoring and microvascular pattern evaluation. Targeted or random biopsies were performed at the antrum, incisura, and corpus, followed by operative link on gastric intestinal metaplasia assessment (OLGIM). The sensitivity, specificity, positive likelihood ratios (PLR), and positive predictive values (PPV) of both EGGIM and the modified EGGIM model for assessing GC risk in GIM patients were validated.

**Results:**

A total of 144 patients were enrolled. The area under curve (AUC) of the receiver operating characteristic of EGGIM for diagnosing OLGIM III-IV was 0.885 (95% CI 0.837–0.943). At the cutoff value ≥4, the sensitivity, specificity, PLR, and PPV were 90.90%, 73%, 3.36, and 50%, respectively. Altered microvascular pattern was identified as a risk factor for high-risk GC development in GIM (*p* < 0.001; odds ratio [OR] = 4.696; 95% CI 2.792–7.896). The modified EGGIM model achieved an AUC of 0.921 (95% CI 0.873–0.968) for identifying OLGIM III-IV; at the cutoff value ≥ 2.5, the sensitivity, specificity, PLR, and PPV were 97%, 66.70%, 2.91, and 46.4%, respectively. After incorporating MV, the AUC of the EGGIM score increased from 0.885 to 0.921, suggesting improved diagnostic performance.

**Conclusions:**

Microvascular pattern serves as a significant independent predictor for OLGIM III-IV. The modified EGGIM demonstrated good diagnostic performance for OLGIM III-IV, suggesting its potential as a non-invasive endoscopic tool for assessing the risk stratification of GC development.

## Introduction

According to the latest global cancer statistics, gastric cancer (GC) ranks fifth worldwide in both incidence and mortality among newly diagnosed cancers, highlighting its status as a major global health threat [1]. Gastric intestinal metaplasia (GIM) is a premalignant condition for GC and is associated with a cumulative risk of gastric carcinogenesis [2,3]. GIM represents an intermediate stage in the Correa cascade, which is a stepwise progression from non-atrophic gastritis to atrophic gastritis, dysplasia, and ultimately adenocarcinoma [4]. As metaplastic intestinal glands provide the cellular substrate for neoplastic development, GIM is associated with a substantial risk of gastric carcinogenesis; evidence indicates that the absolute risk of GC occurrence in GIM patients over a 5-year span is 5.8% [5].

The operative link on gastric intestinal metaplasia assessment (OLGIM) staging system is a clinically applicable tool for risk stratification of GC development in GIM. OLGIM stages III and IV are recognized as “high-risk states” for GC [6]. Early endoscopic recognition and surveillance of GIM is critical for preventing its progression to gastric adenocarcinoma [7]. The endoscopic grading of gastric intestinal metaplasia (EGGIM) scores the extent of GIM [8] and shows good concordance with OLGIM, demonstrating high sensitivity, specificity, and positive likelihood ratio (PLR) for the diagnosis of OLGIM III-IV [9–11]. Beyond mucosal morphology, abnormal angiogenesis is a hallmark of tumorigenesis. The presence of irregular microvascular (MV) pattern is a characteristic endoscopic feature of early GC. Kenshi Yao proposed the MV classification system, categorizing MV pattern as regular, irregular, or absent [12]. MV pattern evolves progressively during the transformation of the gastric mucosa into malignant lesions. Clinical observations revealed that some patients with GIM exhibit concomitant MV abnormalities, even in the absence of a borderline, suggesting that such MV changes may play a crucial role in the risk assessment of gastric carcinogenesis.

Nonetheless, the EGGIM system has not been widely validated and remains scarcely utilized in clinical practice. Therefore, this study aimed to evaluate the applicability and diagnostic accuracy of EGGIM for identifying high-risk GC progression in GIM patients. Notably, the accuracy of the combined assessment of GIM and MV pattern to evaluate the risk of gastric cancer is unclear. Thus, this study establishes a modified model that integrates both the EGGIM and MV pattern, aiming to assess its endoscopic value in the risk stratification of GIM.

## Materials and methods

### Study design and participants

The prospective study recruited patients who underwent gastroscopy and pathological tissue biopsy at Beijing Tiantan Hospital, Henan Provincial Hospital of Traditional Chinese Medicine, and Jiangsu Second Traditional Chinese Medicine Hospital from February 2023 to June 2024. The research protocol was approved by the Medical Ethics Committee of Beijing Tiantan Hospital, Capital Medical University (approval number KY2023-004-02). The study had adhered to the principles stated in the ‘Declaration of Helsinki’. All participating patients signed written informed consent forms prior to examination. The inclusion criteria were: (i) Patients aged 18 years or older with full cognitive capacity who underwent gastroscopy. (ii) Patients with successful gastric histopathological biopsy. Exclusion criteria were: (i) Patients with a history of total or subtotal gastrectomy. (ii) Patients who did not consent to undergo biopsy or multiple-site biopsy. (iii) Patients with insufficient biopsy samples for definitive OLGIM staging. (iv) Patients with spinal/tubular villous lesions and central foveal hyperplasia. (v) Patients with contraindications for biopsy, including ongoing use of antiplatelet agents, anticoagulants, or nonsteroidal anti-inflammatory drugs (NSAIDs), as well as those with bleeding disorders. (vi) Patients with cardiovascular or cerebrovascular disease, acute asthma exacerbation, or comatose or unconscious patients.

## Endoscopic procedures and observation

### Pre-observation preparation

An Olympus GIF-H290Z endoscope (Olympus Medical Systems Corp., Tokyo, Japan) was used to perform a sequential systematic examination, utilizing white light endoscopy (WLE), narrow-band imaging (NBI), and magnifying endoscopy with NBI (ME-NBI) in accordance with standardized gastric examination protocols. Adherent mucus and bile were rinsed with saline before observation, followed by adequate luminal distention to ensure optimal mucosal visualization.

### EGGIM and image documentation

GIM was assessed in real-time by two endoscopists at five gastric sites, including the greater/lesser curvature of antrum, incisura, and the greater/lesser curvature of corpus. According to the EGGIM criteria [9], GIM was classified as follows: no GIM (0 points), GIM ≤30% (1 point), and GIM >30% (2 points). When discordance in GIM extent assessment occurred between endoscopists, a reassessment of the lesion extent was performed for the final score. Both the score for each site and high-definition endoscopic images were documented.

### MV Pattern scoring and documentation

MV pattern were classified according to Kenshi Yao [12]: Regular: The mucosal capillaries have a uniform shape and consistent size, with regular and symmetrical arrangement and distribution; Irregular: The capillaries differ in shape and size, and their arrangement and distribution are irregular and asymmetrical, which may be closed-loop (polygonal), open-loop, tortuous, branched, and bizarrely shaped, with or without a network; Absent: The subepithelial MV pattern is obscured by the presence of an opaque substance within the superficial part of the mucosa. In this study, scores were assigned based on the MV pattern: regular MV was scored as 0, irregular MV as 1, and absence as 2. The morphology of the diseased mucosal microvasculature was scored and photographed under ME-NBI. Scoring and high-definition image documentation were performed under ME-NBI. When observing MV patterns, we deliberately avoided areas such as post-biopsy bleeding, erosion, or ulceration that could interfere with vascular visualization to ensure the accuracy of the assessment. The endoscopists diagnosing microvascular patterns and those performing EGGIM assessments are the same two physicians. Two endoscopists independently assessed the microvascular patterns of all patients without knowledge of each other’s results or the patients’ OLGIM staging.

*Helicobacter pylori* infection and bile reflux: *H. pylori* status was determined based on histological examination and/or the 13 C-urea breath test (UBT). Patients with at least one positive test result at enrollment were classified as having current infection. Patients with documented evidence of prior eradication therapy (based on medical records) and negative histological examination at enrollment were classified as having previous infection. Patients with no history of *H. pylori* infection and negative results on both tests were classified as having no infection. Bile reflux was primarily diagnosed based on the color and consistency of mucus paste observed during esophagogastroduodenoscopy, including pale yellow, yellow-green, or dark yellow mucus paste that is cloudy and viscous, with bile spots visible.

## Biopsies and histopathologic evaluation

Targeted or random biopsy was performed on the above five sites according to the presence or absence of GIM (Supplementary materials). All enrolled patients underwent the recommended biopsy sampling. Biopsy specimens were placed in separate transparent glass containers containing 10% formalin and labeled with the site, number, and name. The histopathologic diagnosis was made by two pathologists who were blinded to the clinical and endoscopic data. Histopathologic results were collected and recorded by the investigator, and OLGIM results were derived and recorded for each site. The histological criteria in atrophy assessment [13,14] are provided in the Supplementary materials.

## Statistical analysis

The sample size required to achieve the study objectives was determined using statistical principles with Power Analysis and Sample Size (PASS). Based on the areas under curve (AUC) of the receiver operating characteristic (ROC) reported in previous study, with value of 0.96 (95% Confidence Interval [CI] 0.93–0.98). With prior studies reporting proportions of OLGIM = 0 and OLGIM ≠ 0 stage as 49.1% vs. 51.9%, 54.4% vs. 45.6%; hence, a GIM patient proportion of 50% was assumed. Considering the significance level (α = 0.05) and statistical power (1–β = 0.9), a total sample size of 140 was calculated.

Analyses were performed using SPSS 25.0, R4.3.2. Descriptive statistics presented continuous variables as mean ± standard deviation (M ± SD) or median (M) with interquartile range (IQR; P25, P75), and categorical variables as frequencies and percentages. Logistic regression was performed for correlation analysis of MV pattern with OLGIM III-IV. ROC curves were used to determine the optimal cutoff values of EGGIM and modified EGGIM model for the diagnosis of OLGIM III-IV. The diagnostic performance was assessed based on the sensitivity, specificity, positive/negative predictive values (PPV/NPV), and positive/negative likelihood ratios (PLR/NLR). Associations between age and different EGGIM scores and OLGIM stages were analyzed by ANOVA or Kruskal-Wallis H test. The Bonferroni-corrected Mann-Whitney U-test revealed significant differences of OLGIM stages. Chi-square test was used for correlations analysis of *Helicobacter pylori* (*H. pylori*) infection rate and bile reflux with different EGGIM scores and OLGIM stages. Statistical significance was defined as two-sided *p* < 0.05.

## Results

## Patient’s clinical characteristics

A total of 144 patients were included in the study. The age range was 29–83 years, with a median age of 58 years. Gastrointestinal symptoms were present in 127 patients (88.19%), among which the main manifestations were epigastric pain (53.47%) and abdominal distension (54.17%), as shown in Table 1.

**Table 1.** Demographic and clinical characteristics, and endoscopic and histological features.

Total patients, n	144
Age, median (IQR), years	58 (46, 64.50)
Gender, n (%)	
Male	92 (63.89)
Female	52 (36.11)
*H. pylori* infection, n (%)	73 (50.69)
Current infection	41 (28.47)
Previous infection	32 (22.22)
Successful eradication of Hp infection, n	36
PPI use (as part of regular medication), n (%)	40 (27.78)
Smoking (current/ex-smoker), n (%)	65 (45.14)
Alcohol (≥ 40 g/day), n (%)	40 (27.78)
Family history of stomach cancer (first- or second-degree relatives), n (%)	3 (2.08)
Adverse events, n (%)	0
Symptoms, n (%)	127 (88.19)
Bile reflux, n (%)	36 (25)
Intestinal metaplasia, n (%)	
Antrum	112 (77.78)
Incisura	105 (72.92)
Corpus	41 (28.47)
Microvascular pattern, n (%)	
Regular	106 (73.61)
Irregular	30 (20.83)
Absent	8 (5.56)
EGGIM score	
EGGIM 0	27 (18.75)
EGGIM 1–2	18 (12.50)
EGGIM 3–4	57 (39.58)
EGGIM 5–7	36 (25.00)
EGGIM 8–10	6 (4.17)

**Table:** 

Histopathology	n (n)/n (%)
Atrophic gastritis (moderate to severe)	
Antrum	24 (10)
Incisura	17 (8)
Corpus	12 (8)
n	41
Intestinal metaplasia (moderate to severe)	
Antrum	105 (56)
Incisura	84 (49)
Corpus	38 (17)
n	116
Dysplasia	
Antrum	11 (52.38)
Incisura	7 (33.33)
Corpus	3 (14.29)
n	15
OLGIM stage	
OLGIM 0	28 (19.44)
OLGIM I	37 (25.69)
OLGIM II	46 (31.94)
OLGIM III	24 (16.67)
OLGIM IV	9 (6.25)
Dysplasia	
OLGIM stage among dysplasia patients	
OLGIM I-II	6 (40.0)
OLGIM III–IV	9 (60.0)
EGGIM score among dysplasia patients	
EGGIM 0–4	5 (33.3)
EGGIM 5–10	10 (66.7)

Current infection was defined as a positive result on either histology or UBT at enrollment. Previous infection was defined as documented eradication history with negative tests at enrollment.

## Endoscopic diagnostic and histologic results

The EGGIM (score range 0–10) results were regrouped into five categories to mitigate distributional bias due to sparse counts (e.g. 1–3 cases) in certain scores, consistent with prior methodologies: EGGIM 0, EGGIM 1–2, EGGIM 3–4, EGGIM 5–7, and EGGIM 8–10. In this study, the predominant scores were EGGIM 0 (18.75%), EGGIM 3–4 (39.58%), and EGGIM 5–7 (25%). Histologically, the cohort comprised 33 patients with atrophic gastritis (AG) with GIM, 68 patients with GIM, and 15 patients with dysplasia, including 8 cases of dysplasia with AG + GIM, and 7 cases of dysplasia with GIM. Anatomic distribution of dysplasia (*n* = 15) was as follows: 1 case involved the antrum, incisura, and corpus; 4 involved the antrum, incisura; 1 involved the incisura and corpus; 6 involved only the antrum; 2 involved the incisura; and 1 involved the corpus, as shown in Table 1. Among the patients with a history of *H. pylori* infection who underwent successful eradication therapy (*n* = 36, Table 1), histological examination revealed 3 cases of normal gastric mucosa, 10 cases of AG with GIM, 18 cases of GIM, 3 cases of AG with IM and dysplasia, and 2 cases of dysplasia with GIM. The quadratic weighted kappa values for inter-observer and intra-observer agreement in microvascular pattern assessment were 0.922 (95% CI 0.769–1.000) and 0.962 (95% CI 0.887–1.000), respectively (Supplementary Table 1).

## EGGIM vs. OLGIM

**Figure 1.**
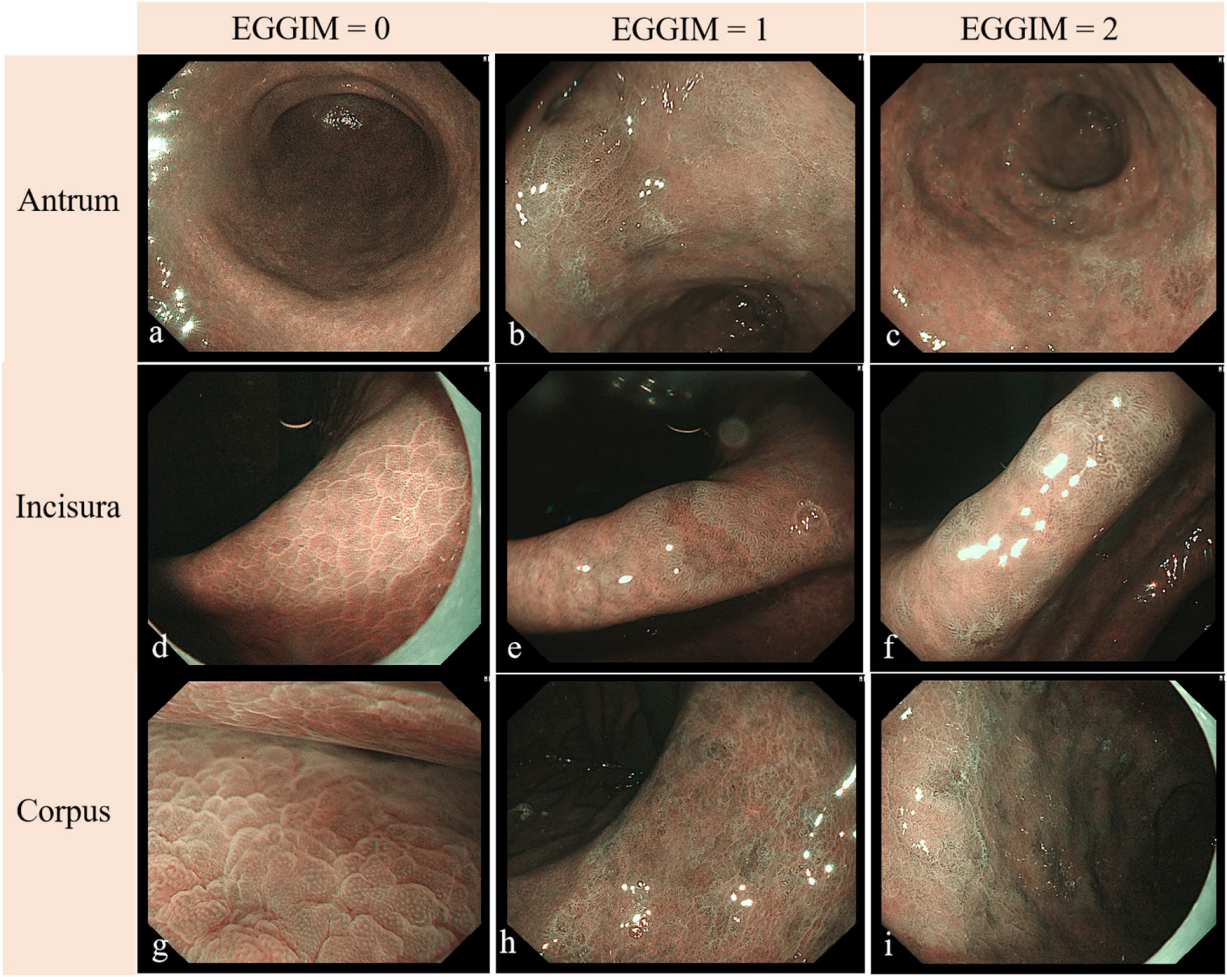
Endoscopic manifestations under NBI across EGGIM scores in the antrum, incisura, and corpus. a, d, g (EGGIM 0): Normal gastric mucosa in the antrum (a), incisura (d), and corpus (g). b, e, h (EGGIM 1): Ridged or villous patterns with light blue crests and/or cryptic fat deposits affecting ≤30% of the mucosal surface in the antrum (b), incisura (e), and corpus (h). c, f, i (EGGIM 2): Extensive ridged/villous patterns (>30% mucosal involvement) with light blue crests and/or cryptic fat deposits in the antrum (c), incisura (f), and corpus (i).

The performance of different EGGIM scores of the antrum, incisura, and corpus is shown in Figure 1. Among OLGIM 0 patients (*n* = 28), 18 patients (64.29%) scored EGGIM 0, and 10 patients (35.71%) scored EGGIM 1–4, including EGGIM1 [*n* = 2, focal GIM at the incisura (1 case) and greater curvature of antrum (1 case)]; EGGIM2 (*n* = 3), focal GIM at antrum; and EGGIM3 (*n* = 5), focal GIM of both the antrum and incisura. The quadratic weighted kappa values for inter-observer and intra-observer agreement in EGGIM assessment were 0.986 (95% CI 0.966–1.000) and 1 (95% CI 1.000-1.000), respectively (Supplementary Table 2). In the OLGIM I-II cohort (*n* = 83), EGGIM 1–4 predominated (65.1%), though 9 cases (10.8%) were graded EGGIM 0 due to undetected focal lesions. Among OLGIM III-IV patients (*n* = 33), EGGIM 5–10 predominated (66.7%, Supplementary Table 3).

## Diagnostic accuracy for OLGIM III-IV across EGGIM cut-offs

**Figure 2.**
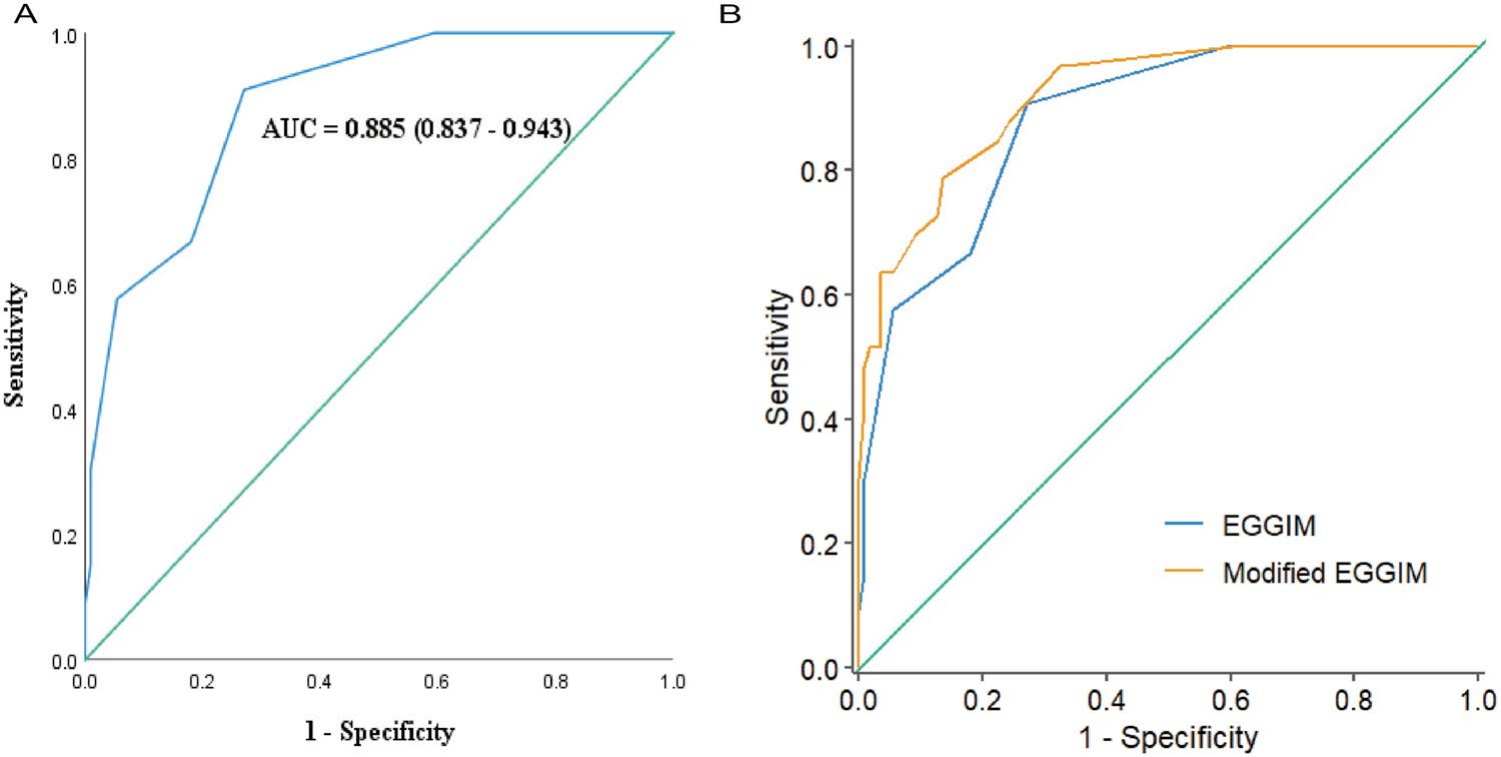
ROC of EGGIM (blue line) for the diagnosis of OLGIM III-IV (2 A). The AUC was 0.885 (*p* < 0.001, 95% CI 0.837–0.943). ROC of modified EGGIM (orange line) and EGGIM (blue line) for the diagnosis of OLGIM III-IV (2B). The AUC (modified EGGIM model) was 0.921 (95% CI 0. 873-0.968). *p* = 0.06.

For the identification OLGIM III-IV, the area under the ROC curve of EGGIM was 0.885 (95% CI 0.837–0.943) (Figure 2A). As shown in Table 2, different cutoffs could be used with high sensitivity, specificity, PPV, NPV, PLR, and NLR. At the maximum Youden index of 0.639, the optimal cutoff was identified as ≥4, yielding balanced diagnostic performance: sensitivity 90.90% (95% CI 81.10–100), specificity 73% (95% CI 64.70–81.20), PLR 3.36 (95% CI 2.43–4.65), PPV 50% (95% CI 37.30–62.70), and NPV 96.40% (95% CI 92.50–100).

**Table 2.** Sensitivity, specificity, PPV, NPV, PLR and NLR of EGGIM at different cutoff values for identifying OLGIM III-IV.

Threshold	Sensitivity	Specificity	PLR	NLR	PPV	NPV
≥1	100 (100–100)	24.30 (16.30–32.30)	1.32 (1.19–1.47)	0 (0-NC)	28.20 (20.10–36.40)	100 (100–100)
≥2	100 (100–100)	27.90 (19.60–36.30)	1.39 (1.24–1.56)	0 (0-NC)	29.20 (20.80–37.60)	100 (100–100)
≥3	100 (100–100)	40.50 (31.40–49.70)	1.68 (1.44–1.96)	0 (0-NC)	33.30 (24.00–42.60)	100 (100–100)
≥4	90.90 (81.10–100)	73 (64.70–81.20)	3.36 (2.43–4.65)	0.13 (0.04–0.37)	50 (37.30–62.70)	96.40 (92.50–100)
≥5	66.70 (50.60–82.80)	82.00 (74.80–89.10)	3.70 (2.33–5.89)	0.41 (0.25–0.66)	52.40 (37.30–67.50)	89.20 (83.20–95.20)
≥6	57.60 (40.70–74.40)	94.60 (90.40–98.80)	10.65 (4.64–24.47)	0.45 (0.30–0.67)	76 (59.30–92.70)	88.20 (82.40-94)
≥7	30.30 (14.60-46)	99.10 (97.30–100)	33.64 (4.47–253.21)	0.70 (0.56–0.88)	90.90 (73.90–100)	82.70 (76.30–89.10)
≥8	15.20 (2.90–27.40)	99.10 (97.30–100)	16.82 (2.04–138.90)	0.86 (0.74–0.99)	83.30 (53.50–100)	79.70 (73-86.40)
≥9	9.10 (−0.70-18.90)	100 (100–100)	Inf (NC-Inf)	0.91 (0.82–1.00)	100 (100–100)	78.70 (72-85.50)
10	3 (−2.80-8.90)	100 (100–100)	Inf (NC-Inf)	0.97 (0.91–1.00)	100 (100–100)	77.60 (70.80–84.50)

Positive/negative predictive values, PPV/NPV; positive/negative likelihood ratios, PLR/NLR. NC (not calculable), cannot be calculated. Inf (infinity), reflects the characteristic of “PLR at 100% specificity”.

## Establishment of the modified EGGIM model

Logistic regression revealed a significant association between MV and OLGIM III-IV, with a regression coefficient of 1.547 (*p* < 0.001), indicating predictive value. The OR was 4.696 (95% CI 2.792–7.896) and model fit was established by the Hosmer-Lemeshow test (χ^2^ = 2.129, *p* = 0.145) (Supplementary Table 4).

**Figure 3.**
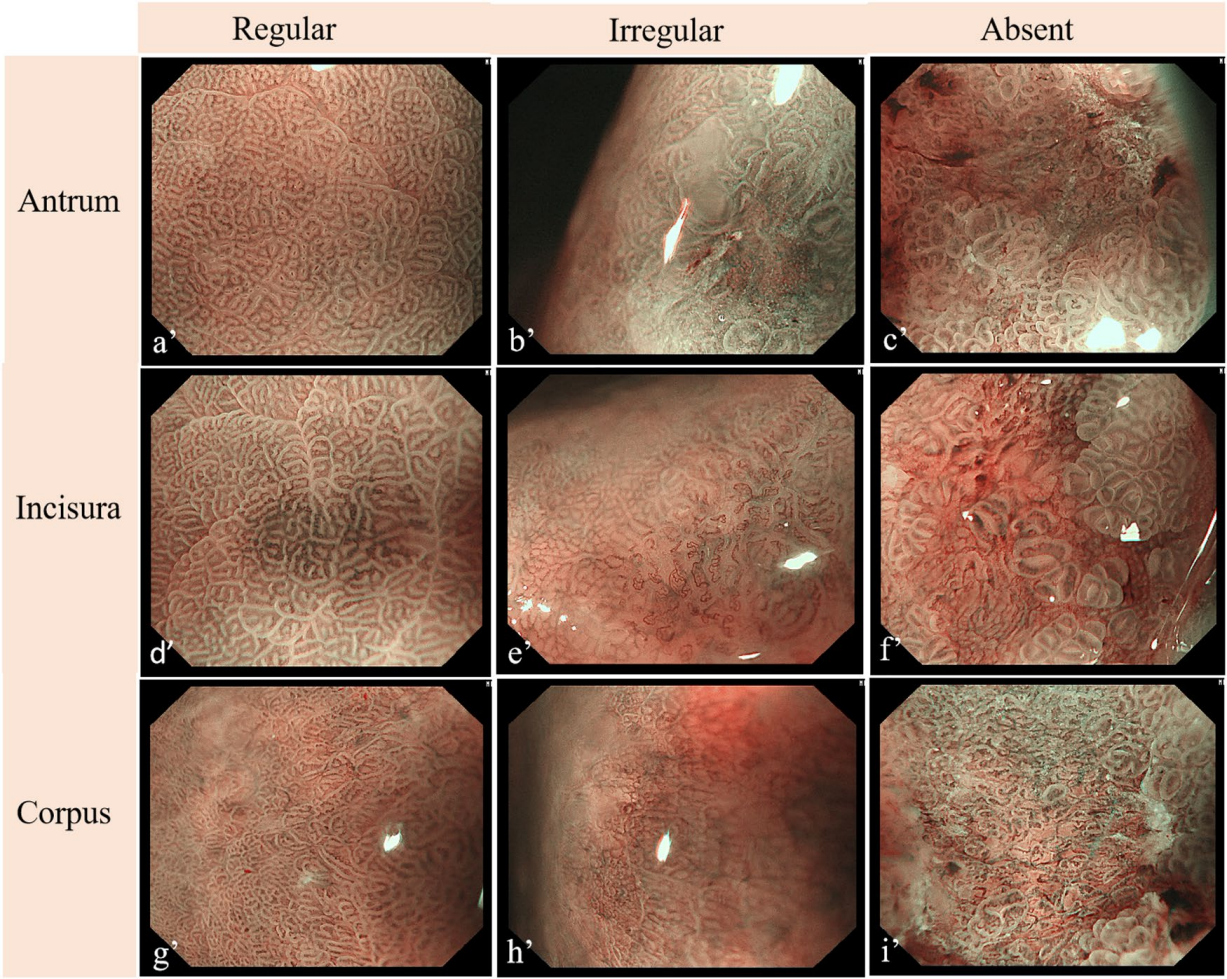
Endoscopic manifestations of MV under NBI for the modified EGGIM model in the antrum, incisura, and corpus. a’, d’, g’ demonstrate regular MV in the antrum (a’), incisura (d’), and corpus (g’). b’, e’, h’ show irregular MV including dilation, tortuosity, and heterogeneity in the antrum (b’), incisura (e’), and corpus (h’). c’, f’, i’ exhibit absence of MV in the antrum (c’), incisura (f’), and corpus (i’).

Scoring criteria for the modified EGGIM model integrating MV pattern were defined as follows: absence of GIM (0 point), focal GIM (≤30% mucosal involvement, 1 point), extensive GIM (>30%, 2 points); regular microvasculature (0 point), irregular (1 point), absent (2 points). Multivariable logistic regression in 144 patients from three centers produced β-coefficients of 0.783 (EGGIM) and 1.155 (MV), establishing the diagnostic algorithm. To facilitate clinical adoption, we have simplified the complex calculations based on regression coefficients into an intuitive scoring system: Modified Score = 0.8 × (EGGIM) + 1.2 × MV. Endoscopic manifestations of MV are shown in Figure 3.

## Modified EGGIM model and OLGIM

Among 28 patients with OLGIM 0, 26 (92.9%) had a modified EGGIM score of 0. For OLGIM I-II cases, the modified EGGIM scores concentrated in the (1, 3.5) range (59.04%). In OLGIM III-IV, scores predominantly fell within [3.5, 7) (48.49%) and [7, 10.512] (39.39%) intervals. Across the entire cohort, 18.06% of patients had a modified EGGIM score of 0, 44.44% fell within the range (0, 3.5), 27.78% within [3.5, 7), and 9.72% within [7, 10.512] (Supplementary Table 5).

## Accuracy estimates for the diagnosis of OLGIM III-IV using different modified EGGIM score cut-offs

Using OLGIM III-IV as the benchmark for high-risk GC progression, the modified EGGIM model achieved an area under the ROC curve of 0.921 (95% CI 0.873–0.968) (Figure 2B). At the optimal cutoff of 2.5, balanced diagnostic performance was observed: sensitivity 97% (95% CI 91.10–100), specificity 66.70% (95% CI 57.90–75.40), PLR 2.91 (95% CI 2.22–3.81), and NPV 98.70% (95% CI 96.10–100), as shown in the Table 3. After incorporating MV, the predictive capability of the EGGIM score improved (AUC increased from 0.885 to 0.921).

**Table 3.** Sensitivity, specificity, PPV, NPV, PLR and NLR of modified EGGIM with different cutoff values for assessment of OLGIM III-IV.

Threshold	Sensitivity	Specificity	PLR	NLR	PPV	NPV
≥0.5	100 (100–100)	23.40 (15.50–31.30)	1.31 (1.18–1.45)	0 (0-NC)	28 (19.90–36.10)	100 (100–100)
≥1.5	100 (100–100)	27 (18.80–35.30)	1.37 (1.22–1.54)	0 (0-NC)	28.90 (20.60–37.30)	100 (100–100)
≥2.5	97 (91.10–100)	66.70 (57.90–75.40)	2.91 (2.22–3.81)	0.045 (0.007–0.32)	46.40 (34.60–58.10)	98.70 (96.10–100)
≥3.5	87.90 (76.70-99)	75.70 (67.70–83.70)	3.61 (2.54–5.14)	0.16 (0.06–0.40)	51.80 (38.70–64.90)	95.50 (91.10–99.80)
≥4.5	72.70 (57.50–87.90)	87.40 (81.20–93.60)	5.77 (3.39–9.82)	0.31 (0.18–0.55)	63.20 (47.80–78.50)	91.50 (86.20–96.80)
≥5.5	57.60 (40.70–74.40)	96.40 (92.90–99.90)	15.98 (5.84–43.68)	44 (0.30–0.66)	82.60 (67.10–98.10)	88.40 82.70–94.10)
≥6.5	48.50 (31.40–65.50)	99.10 (97.30–100)	53.82 (7.41–390.80)	52 (0.37–0.72)	94.10 (82.90–100)	86.60 (80.70–92.50)
≥7.5	27.30 (12.10–42.50)	100 (100–100)	Inf (NC-Inf)	72.70 (0.59–0.90)	100 (100–100)	82.20 (75.80–88.70)
≥8.5	15.20 (2.90–27.40)	100 (100–100)	Inf (NC-Inf)	84.80 (0.74–0.98)	100 (100–100)	79.90 (73.20–86.50)
≥9.5	9.10 (−0.70-18.90)	100 (100–100)	Inf (NC-Inf)	90.90 (0.82–1.00)	100 (100–100)	78.70 (72-85.50)
≥10.5	6.10 (−2.10-14.20)	100 (100–100)	Inf (NC-Inf)	93.90 (0.86–1.00)	100 (100–100)	78.20 (71.40-85)

Positive/negative predictive values, PPV/NPV; positive/negative likelihood ratios, PLR/NLR. NC (not calculable), cannot be calculated. Inf (infinity), reflects the characteristic of “PLR at 100% specificity”.

## Analysis of the association of age, *H. pylor*i infection, and bile reflux with EGGIM

Age distribution differed significantly across the different EGGIM score groups (*p* = 0.032). Further post-hoc comparisons by Holm-Bonferroni correction revealed a statistically significant difference between EGGIM 0 and EGGIM 8–10 groups (*p* = 0.05). Both mean and median age increased with higher EGGIM scores. *H. pylori* infection rates varied significantly among EGGIM score groups (*p* = 0.041, Pearson’s chi-square test), demonstrating a progressive increase from EGGIM 0 to EGGIM 7. In contrast, no significant association was observed between bile reflux and EGGIM scores (*p* = 0.761, chi-square test), as shown in Table 4.

**Table 4.** The association of age, *H. pylori* infection, and bile reflux with EGGIM.

EGGIM	0	1–2	3–4	5–7	8–10	H/χ^2^	*P*
n	27	18	57	36	6		
Age (M ± SD)	50 ± 12.56	56.28 ± 12.73	56.37 ± 11.24	57.11 ± 11.64	65.67 ± 7.12	10.55	0.032*
*H. pylori* infection, n (%)	10 (37.04)	7 (38.89)	27 (47.37)	26 (72.22)	3 (50)	9.947	0.041*
Bile reflux, n (%)	5 (18.52)	3 (16.67)	16 (28.07)	10 (27.78)	2 (33.33)	2.014	0.761

* : *p* < 0.05, the difference is statistically significant.

## Analysis of the association of age, *H. pylor*i infection, and bile reflux with OLGIM

A statistically significant difference was found in the age distribution of patients across the OLGIM 0-IV (*p* = 0.011). Post-hoc analysis with the Bonferroni-corrected Mann-Whitney U-test revealed significant differences between OLGIM 0 and I, and between OLGIM 0 and IV (Adjusted *p* = 0.04). Chi-square test showed a significant association between OLGIM and *H. pylori* infection (*p* = 0.046). There was no statistically significant difference in bile reflux prevalence across OLGIM 0-IV (*p* > 0.05), and the Cochran-Armitage trend test showed that bile reflux increased with the OLGIM stages. The Cochran-Armitage trend test indicated no significant trend with increasing OLGIM stages (*Z* = 1.46, *p* = 0.14). Although each stage increases in OLGIM showed a non-significant 15% elevated bile reflux risk (OR per stage = 1.15; 95% CI 0.95–1.40; *p* = 0.15), stage IV exhibited a trend toward higher risk (OR = 2.22) (Table 5).

**Table 5.** The association of age, *H. pylori* infection, and bile reflux with OLGIM.

OLGIM	0	I	II	III	IV	H/χ^2^	*P*
n	28	37	46	24	9		
Age (M ± SD, Median (IQR))	50.89 ± 11.16	59.65 ± 11.95	54.96 ± 11.98	56.21 ± 11.80	63 (61, 66)	13.131	0.011*
*H. pylori* infection, n (%)	9 (32.14)	18 (48.65)	23 (50.00)	18 (75.00)	5 (55.56)	9.684	0.046*
Bile reflux, n (%)	6 (21.43)	6 (16.22)	14 (30.43)	6 (25.00)	4 (44.44)	4.252	0.373

* : *p* < 0.05, the difference is statistically significant.

## Discussion

The identification, surveillance, and intervention of gastric precancerous lesions constitute a critical component of GC management strategies. Endoscopic risk stratification for GC development in patients with GIM holds significant clinical value. Our prospective study validates the diagnostic accuracy and clinical utility of the EGGIM for identifying high-risk GIM patients (OLGIM III-IV). Alterations in MV pattern serve as significant predictors of OLGIM III-IV.

The study confirms strong concordance between EGGIM and OLGIM in assessing the severity of GIM, with rising EGGIM scores correlating with advanced OLGIM stages. The diagnostic performance of EGGIM (AUC 0.885, 95% CI 0.837–0.943; cutoff ≥4) aligns with previous reports from European (AUC 0.96, 95% CI 0.93–0.98; cutoff >4) [9] and Chinese cohorts (AUC 0.949, 95% CI 0.916–0.972; cutoff ≥4) [11], supporting its reliability as an endoscopic risk stratification tool across diverse populations.

Malignant transformation of the gastric mucosa is accompanied by characteristic MV changes, with irregular patterns serving as endoscopic hallmarks of early GC [12]. Given that white opaque substance (WOS) may obscure microsurface assessment under white-light imaging (WLI) [15,16], this study focused on MV pattern evaluation, a parameter assessable under both WLI and NBI, to avoid redundant WOS assessment and enhance feasibility. Compared with noncancerous tissues, the MV pattern of cancerous tissues exhibits irregularity characterized endoscopically by vasodilation, abrupt caliber changes, tortuosity, heterogeneous caliber, variable morphology, and absence of vessels [17,18]. Correlation analysis revealed a significant independent association between MV scores and OLGIM III–IV (Hosmer–Lemeshow test, *p* = 0.145), identifying MV abnormality as a key endoscopic feature for risk stratification in GIM patients.

Building on this finding, this study established a modified EGGIM model incorporating with EGGIM and MV pattern. This modified model achieved an AUC of 0.921 (95% CI 0.873–0.968) for diagnosing OLGIM III–IV, with a sensitivity of 97.0% (95% CI 91.10–100) and specificity of 66.7% (95% CI 57.9–75.4) at an optimal cutoff of ≥2.5. This study further compared the diagnostic efficacy of EGGIM and modified EGGIM model for diagnosing OLGIM III-IV. The modified EGGIM model showed a higher AUC value and sensitivity than EGGIM. Although the boundary of statistical significance was not reached (*p* = 0.06), the improved discrimination demonstrated by modified EGGIM model has potential clinical value. This implies that in clinical practice, particularly in high-risk population screening where diagnostic accuracy is paramount, the modified model has the potential to identify additional patients who would be missed by conventional methods. It offers a more comprehensive assessment system that integrates mucosal alterations with microcirculatory changes.

The onset, progression, and severity of GIM represent a complex pathological process. This study further examined the associations of age, *H. pylori* infection, and bile reflux with EGGIM and OLGIM to provide a more comprehensive clinical basis for individualized risk management. GIM is an age-related condition characterized by exponential accumulation of senescent cells in local tissues with advancing chronological age, leading to tissue dysfunction [19–22]. Our analysis revealed statistically significant differences in age distribution across EGGIM score groups, with a trend of increasing median age corresponding to higher EGGIM scores. Furthermore, age demonstrated a positive association with advancing OLGIM stages, consistent with population-based study from South Korea [23] and supports the concept that patients aged ≥63 years with OLGIM IV may need shorter follow-up intervals.

*H. pylori* infection prevalence correlates with GIM [24–26]. In this study, *H. pylori* infection rates were significantly associated with both EGGIM and OLGIM 0-IV. We observed a progressive increase in *H. pylori* infection rates corresponding to higher EGGIM scores (0–7 points) and elevated OLGIM stages (I-III). Evidence from prior studies confirmed that *H. pylori* eradication induces regression of AG and stabilizes mild GIM, ultimately reducing progression to GC. Hence, early *H. pylori* eradication in GIM patients maximizes clinical benefit [27–29].

Bile reflux is a known risk factor for chronic gastritis and gastric mucosal injury, potentially promoting the occurrence and progression of intestinal metaplasia in the gastric mucosa. Bile reflux constitutes a recognized contributor to GIM development [30–32]. However, its associations with EGGIM and OLGIM remain underexplored. Our study found no statistically significant correlation between bile reflux and either EGGIM or OLGIM although the highest proportions of bile reflux patients were found in those with EGGIM 8–10 and OLGIM IV. This observation suggests bile reflux may serve as a potential contributor to progressive EGGIM/OLGIM escalation, though the lack of statistical significance and inability to definitively ascertain its temporal onset and severity warrant cautious interpretation. Rigorous prospective studies are needed to further elucidate this relationship.

Our study has several limitations. First, the diagnostic rate of GIM in esophagogastroduodenoscopy was higher than histopathology results, which may be attributed to the insufficient biopsy depth and small tissue size of some biopsies, limiting pathological assessment. Second, the sample size assumption was optimistic relative to the observed diagnostic performance of EGGIM, and external validation is needed. Third, the modified EGGIM model represents a preliminary exploration; its clinical application value and significance need to be validated through large-scale multicenter studies, its stability and generalizability need to be confirmed in independent external cohorts. Additionally, while *H. pylori* infection rate correlated with GIM severity, its non-linear relationship with advancing EGGIM scores and OLGIM stages may stem from limited number of patients with EGGIM scores above 8 and OLGIM IV. Last, the limited sample size in the OLGIM IV subgroup resulted in underpowered exploratory subgroup analyses of age, *H. pylori* infection, and bile reflux. It should be noted that the intra-observer weighted kappa for EGGIM reached 1.000, indicating perfect agreement. This finding should be interpreted with caution, as the 4-week re-evaluation interval may not be sufficient to fully exclude recall bias. Future validation studies should employ a longer interval between assessments and explicitly document the blinding procedure to minimize potential bias.

In summary, the findings indicate the clinical applicability of EGGIM for identifying GIM patients at high risk of GC. The model combining MV and EGGIM has considerable value in identifying GIM patients at high risk for GC, which may provide additional endoscopic visualization tools for clinical practice. The modified EGGIM model developed in this study lies in providing a refined risk stratification tool. By integrating dual information from mucosal glands and microvasculature, it may be beneficial for enhancing endoscopic monitoring and follow-up of patients with a high risk for GC, and preventing progression to high-grade intraepithelial neoplasia and early GC.

## Supplementary Material

Supplemental Material

Clean Supplementary materials.docx

## Data Availability

The data can be requested from the corresponding author.

## References

[1] Bray F, Laversanne M, Sung H, et al. Global cancer statistics 2022: GLOBOCAN estimates of incidence and mortality worldwide for 36 cancers in 185 countries. CA Cancer J Clin. 2024;74(3):229–263. doi: 10.3322/caac.21834. 38572751

[2] Lee JWJ, Zhu F, Srivastava S, et al. Severity of gastric intestinal metaplasia predicts the risk of gastric cancer: a prospective multicentre cohort study (GCEP). Gut. 2022;71(5):854–863. doi: 10.1136/gutjnl-2021-324057. 33975867 PMC8995828

[3] Yue SSK, Tong Y, Siu HC, et al. Divergent lineage trajectories and genetic landscapes in human gastric intestinal metaplasia organoids associated with early neoplastic progression. Gut. 2025;74(4):522–538. doi: 10.1136/gutjnl-2024-332594. 39572083

[4] Correa P, Haenszel W, Cuello C, et al. A model for gastric cancer epidemiology. Lancet. 1975;2(7924):58–60. doi: 10.1016/s0140-6736(75)90498-5. 49653

[5] Pimentel-Nunes P, Libânio D, Marcos-Pinto R, II, et al. Management of epithelial precancerous conditions and lesions in the stomach (MAPS.): european Society of Gastrointestinal Endoscopy (ESGE), European Helicobacter and Microbiota Study Group (EHMSG), European Society of Pathology (ESP), and Sociedade Portuguesa de Endoscopia Digestiva (SPED) guideline update 2019. Endoscopy. 2019;51(4):365–388. doi: 10.1055/a-0859-1883. 30841008

[6] Dinis-Ribeiro M, Shah S, El-Serag H, et al. The road to a world-unified approach to the management of patients with gastric intestinal metaplasia: a review of current guidelines. Gut. 2024;73(10):1607–1617. doi: 10.1136/gutjnl-2024-333029. 39122364

[7] Morgan DR, Corral JE, Li D, et al. ACG Clinical Guideline: diagnosis and management of gastric premalignant conditions. Am J Gastroenterol. 2025;120(4):709–737. doi: 10.14309/ajg.0000000000003350. 40072510 PMC13166553

[8] Pimentel-Nunes P, Libânio D, Lage J, et al. A multicenter prospective study of the real-time use of narrow-band imaging in the diagnosis of premalignant gastric conditions and lesions. Endoscopy. 2016;48(8):723–730. doi: 10.1055/s-0042-108435. 27280384

[9] Esposito G, Pimentel-Nunes P, Angeletti S, et al. Endoscopic grading of gastric intestinal metaplasia (EGGIM): a multicenter validation study. Endoscopy. 2019;51(6):515–521. doi: 10.1055/a-0808-3186. 30577062

[10] Marcos P, Brito-Gonçalves G, Libânio D, et al. Endoscopic grading of gastric intestinal metaplasia on risk assessment for early gastric neoplasia: can we replace histology assessment also in the West? Gut. 2020;69(10):1762–1768. doi: 10.1136/gutjnl-2019-320091. 32051208

[11] Zhang G, Zheng J, Zheng L, et al. Gastric intestinal metaplasia assessment between linked color imaging based on endoscopy and pathology. Scand J Gastroenterol. 2021;56(1):103–110. doi: 10.1080/00365521.2020.1849385. 33232631

[12] Yao K, Anagnostopoulos GK, Ragunath K. Magnifying endoscopy for diagnosing and delineating early gastric cancer. Endoscopy. 2009;41(5):462–467. doi: 10.1055/s-0029-1214594. 19418401

[13] Rugge M, Correa P, Di Mario F, et al. OLGA staging for gastritis: a tutorial. Dig Liver Dis. 2008;40(8):650–658. doi: 10.1016/j.dld.2008.02.030. 18424244

[14] Capelle LG, de Vries AC, Haringsma J, et al. The staging of gastritis with the OLGA system by using intestinal metaplasia as an accurate alternative for atrophic gastritis. Gastrointest Endosc. 2010;71(7):1150–1158. doi: 10.1016/j.gie.2009.12.029. 20381801

[15] Li Z, Zuo XL, Yu T, et al. Confocal laser endomicroscopy for in vivo detection of gastric intestinal metaplasia: a randomized controlled trial. Endoscopy. 2014;46(4):282–290. doi: 10.1055/s-0033-1359215. 24473908

[16] Wei N, Mulmi Shrestha S, Shi RH. Markers of gastric intestinal metaplasia under digital chromoendoscopy: systematic review and meta-analysis. Eur J Gastroenterol Hepatol. 2021;33(4):470–478. doi: 10.1097/MEG.0000000000001834. 32675780

[17] Muto M, Yao K, Kaise M, et al. Magnifying endoscopy simple diagnostic algorithm for early gastric cancer (MESDA-G). Dig Endosc. 2016;28(4):379–393. doi: 10.1111/den.12638. 26896760

[18] Doyama H, Nakanishi H, Yao K. Image-enhanced endoscopy and its corresponding histopathology in the stomach. Gut Liver. 2021;15(3):329–337. doi: 10.5009/gnl19392. 32200589 PMC8129655

[19] Mansuri I, Goldsmith JD, Liu E, et al. Gastric intestinal metaplasia in children: natural history and clinicopathological correlation. J Pediatr Gastroenterol Nutr. 2023;77(3):332–338. doi: 10.1097/MPG.0000000000003862. 37319118

[20] Simko V, Anand N, Ginter E. Gastric intestinal metaplasia - age, ethnicity and surveillance for gastric cancer. Bratisl Lek Listy. 2015;116(1):3–8. doi: 10.4149/bll_2015_001. 25666954

[21] Horvath S. DNA methylation age of human tissues and cell types. Genome Biol. 2013;14(10):R115. doi: 10.1186/gb-2013-14-10-r115. 24138928 PMC4015143

[22] Hannum G, Guinney J, Zhao L, et al. Genome-wide methylation profiles reveal quantitative views of human aging rates. Mol Cell. 2013;49(2):359–367. doi: 10.1016/j.molcel.2012.10.016. 23177740 PMC3780611

[23] Nam JH, Choi IJ, Kook MC, et al. OLGA and OLGIM stage distribution according to age and Helicobacter pylori status in the Korean population. Helicobacter. 2014;19(2):81–89. doi: 10.1111/hel.12112. 24617667

[24] Kamada T, Haruma K, Inoue K, et al. [Helicobacter pylori infection and endoscopic gastritis -Kyoto classification of gastritis]. Nihon Shokakibyo Gakkai Zasshi. 2015;112(6):982–993. doi: 10.11405/nisshoshi.112.982. 26050720

[25] Zhang Y, Weck MN, Schöttker B, et al. Gastric parietal cell antibodies, Helicobacter pylori infection, and chronic atrophic gastritis: evidence from a large population-based study in Germany. Cancer Epidemiol Biomarkers Prev. 2013;22(5):821–826. doi: 10.1158/1055-9965.EPI-12-1343. 23456556

[26] Sugano K, Moss SF, Kuipers EJ. Gastric intestinal metaplasia: real culprit or innocent bystander as a precancerous condition for gastric cancer? Gastroenterology. 2023;165(6):1352–1366.e1. doi: 10.1053/j.gastro.2023.08.028. 37652306

[27] Akpoigbe K, Culpepper-Morgan J, Nwankwo O, et al. Predicting gastric intestinal metaplasia in a high-risk population. Cureus. 2022;14(11):e31502. doi: 10.7759/cureus.31502. 36532909 PMC9750236

[28] Shah SC, Piazuelo MB, Kuipers EJ, et al. AGA clinical practice update on the diagnosis and management of atrophic gastritis: expert review. Gastroenterology. 2021;161(4):1325–1332.e7. doi: 10.1053/j.gastro.2021.06.078. 34454714 PMC8740554

[29] Choi AY, Strate LL, Fix MC, et al. Association of gastric intestinal metaplasia and East Asian ethnicity with the risk of gastric adenocarcinoma in a U.S. population. Gastrointest Endosc. 2018;87(4):1023–1028. doi: 10.1016/j.gie.2017.11.010. 29155082

[30] Yu JH, Zheng JB, Qi J, et al. Bile acids promote gastric intestinal metaplasia by upregulating CDX2 and MUC2 expression via the FXR/NF-κB signalling pathway. Int J Oncol. 2019;54(3):879–892. doi: 10.3892/ijo.2019.4692. 30747230 PMC6365039

[31] Matsuhisa T, Arakawa T, Watanabe T, et al. Relation between bile acid reflux into the stomach and the risk of atrophic gastritis and intestinal metaplasia: a multicenter study of 2283 cases. Dig Endosc. 2013;25(5):519–525. doi: 10.1111/den.12030. 23363381

[32] Tatsugami M, Ito M, Tanaka S, et al. Bile acid promotes intestinal metaplasia and gastric carcinogenesis. Cancer Epidemiol Biomarkers Prev. 2012;21(11):2101–2107. doi: 10.1158/1055-9965.EPI-12-0730. 23010643

