# Supplementary material for: Endoscopic grading of gastric intestinal metaplasia and microvascular pattern for assessing gastric cancer risk: a prospective study
Source: Ann Med. 2026 May 10;58(1):2668887. doi: 10.1080/07853890.2026.2668887 (PMC13159589; doi:10.1080/07853890.2026.2668887)
Supplement: Supplemental Material [file IANN_A_2668887_SM6184.docx]

Supplementary Table 1. The inter- and intra-observer consistency in the MV score.

| MV score | Weighted kappa | 95% CI |
| --- | --- | --- |
| Inter-observer consistency | 0.922 | 0.769 - 1.000 |
| Tntra-observer consistency | 0.962 | 0.887 - 1.000 |

Supplementary Table 2. The inter- and intra-observer consistency in the EGGIM score.

| EGGIM score | Weighted kappa | 95% CI |
| --- | --- | --- |
| Inter-observer consistency | 0.986 | 0.966 - 1.000 |
| Tntra-observer consistency | 1 | 1.000 - 1.000 |

Supplementary Table 3. Comparison of EGGIM and OLGIM.

|  |  | EGGIM | | |
| --- | --- | --- | --- | --- |
|  |  | 0 | 1-4 | 5-10 |
| GIM |  | Absent | Focal | Extensive |
| OLGIM | 0 stage | 18 (64.29 %) | 10 (35.71 %) | 0 |
|  | I-II stage | 9 (10.84 %) | 54 (65.06 %) | 20 (24.10 %) |
|  | III-IV stage | 0 | 11 (33.33 %) | 22 (66.67 %) |

Supplementary Table 4. Correlation analysis of microvascular pattern, and EGGIM with OLGIM III-IV.

| Variable | β | OR (95% CI) | *P* |
| --- | --- | --- | --- |
| EGGIM | 0.783 | 2.188 (1.517-3.156) | < 0.001 |
| microvascular pattern | 1.155 | 3.175 (1.728-5.834) | < 0.001 |

Supplementary Table 5. Modified EGGIM model and OLGIM.

|  | |  | Modified EGGIM | | | | |
| --- | --- | --- | --- | --- | --- | --- | --- |
|  |  | | 0 | (0, 3.5) | [3.5, 7) | [7, 10.512] | Total |
| OLGIM | 0 stage | | 17 (60.71 %) | 11 (39.29 %) | 0 | 0 | 28 |
|  | I-II stage | | 9 (10.84 %) | 49 (59.04 %) | 24 (28.92 %) | 1 (1.20 %) | 83 |
|  | III-IV stage | | 0 | 4 (12.12 %) | 16 (48.49 %) | 13 (39.39 %) | 33 |
| Total |  | | 26 | 64 | 40 | 14 | 144 |

[ ) denotes a left-closed, right-open interval.
