# Supplementary material for: Endoscopic grading of gastric intestinal metaplasia and microvascular pattern for assessing gastric cancer risk: a prospective study
Source: Ann Med. 2026 May 10;58(1):2668887. doi: 10.1080/07853890.2026.2668887 (PMC13159589; doi:10.1080/07853890.2026.2668887)
Supplement: Clean Supplementary materials.docx [file IANN_A_2668887_SM7496.docx]

Methods

***Atrophy***. Histological assessments were performed on biopsy specimens obtained from the antrum, incisura, and corpus according to the Updated Sydney System. Atrophic gastritis was defined as the loss of appropriate glands, with or without metaplasia. The severity of atrophy was graded as none, mild, moderate, or severe based on the percentage of glandular loss (mild: 1-30%; moderate: 31-60%; severe: > 60%). According to the Updated Sydney System and the OLGA/OLGIM staging systems^[13, 14]^, the histological grade for each patient was determined by the most severe atrophy grade observed in the biopsy sites (antrum, incisura angularis, and corpus). That is, when atrophy is present in multiple gastric regions simultaneously, patients are classified according to the highest atrophy grade among all evaluated sites.

***Biopsies.*** Biopsy was performed strictly according to the updated Sydney System to ensure reliable OLGIM staging, with five standard biopsy specimens obtained from each patient: two from the antrum (one from the greater curvature and one from the lesser curvature, within 2-3 cm of the pylorus), one from the incisura angularis, and two from the corpus (one from the greater curvature and one from the lesser curvature). For sites where GIM was endoscopically visible (e.g., suspicious lesions), targeted biopsies were taken, whereas for sites without visible GIM, random biopsies were taken from the standard locations mentioned above, ensuring that all five sites are evaluated—the cornerstone of accurate OLGIM staging.

***The inter- and intra-observer consistency in the MV score.*** The two endoscopists independently evaluated the microvascular patterns of all patients, blinded to each other's results and to the patients' clinical data. For intra-observer consistency, a random sample of 30 patients was selected, and the same two endoscopists re-evaluated the MV patterns after a 4-week interval, blinded to their previous assessments. The level of agreement was assessed using weighted kappa, with 95% confidence intervals. The inter- and intra-observer consistency in the EGGIM score was assessed using the same methodology.
